# Lactic Acid Bacteria: Variability Due to Different Pork Breeds, Breeding Systems and Fermented Sausage Production Technology

**DOI:** 10.3390/foods9030338

**Published:** 2020-03-13

**Authors:** Giuseppe Comi, Alessia Muzzin, Mirco Corazzin, Lucilla Iacumin

**Affiliations:** Department of Agriculture, Food, Environmental and Animal Science, University of Udine, via Sondrio 2/A, 33100 Udine, Italy

**Keywords:** lactic acid bacteria, fermented sausages, ecology, breed, breeding system

## Abstract

Changes in the ecology of the various lactic acid bacteria (LAB) species, which are involved in traditional fermented sausages, were investigated in the light of the use of different breeds of pork, each of which was raised in two different environments and processed using two different technologies. The semi-quantitative molecular method was applied in order to understand how the different species alternate over time, as well as their concentration ratios. A significant increase in LAB over the first days of fermentation characterized the trials where the starter culture wasn’t added (T), reaching values of 10^7^–10^8^ cfu g^−1^. On the other hand, in the trials in which sausages were produced with starter addition, LAB counts had a less significant incremental jump from about 10^6^ cfu g^−1^ (concentration of the inoculum) to 10^8^ cfu g^−1^. *Lactobacillus sakei* and *Lb. curvatus* were detected as the prevalent population in all the observed fermentations. *Pediococcus pentosaceus, Lb. casei, Leuconostoc mesenteroides, Lactococcus garviae*, and *Lb. graminis* also appeared, but their concentration ratios varied depending on the diverse experimental settings. The results of cluster analysis showed that a plant- and breed-specific LAB ecology exists. In addition, it was also observed that the breeding system can influence the presence of certain LAB species.

## 1. Introduction

Traditional fermented sausages are well-known and popular meat products in Italy. In north-eastern Italy, Friuli Venezia Giulia region, traditional fermented sausages are made without the use of microbial starters to preserve the typical characteristics of these products, that are delicate sourness gentle acidity, and elastic consistency. These foodstuffs are made from about 60% of minced fresh pork meat and 40% of minced lard mixed with sugar, NaCl, spices and additives (i.e., nitrate, nitrite). Starter cultures are used mainly for large-scale productions [1,2,3].

The study of microbial ecology during the fermentation of cured meat products began in the 70s [4]. Since then, fifty years of studies have shown that the two groups responsible for the characteristic transformations of these products, regardless of the specific technologies and recipes applied in the different regional cultures, are lactic acid bacteria (LAB) and coagulase-negative catalase-positive cocci (CNCPC) [5,6,7,8,9]. Among them, the species that develop are closely related to the process conditions applied. Specifically, the first parameter of selection is the temperature, which selectively favours the development of some LAB species at the expense of others during the first days of ripening. Sausages subjected to a short ripening using high ripening temperature allow the development of lactobacilli right from the early stages of fermentation and, at the end of ripening, an acidic flavour with slight aroma predominates in salami. Indeed, the pH decreases due to the transformation of sugars caused by the high concentration of lactobacilli and consequently inhibits the growth of CNCPC [9,10,11,12].

This phenomenon is directly proportional to the increase in temperature, in fact, the higher the temperature the more the selection pushes towards more acidifying species, with an increasingly more rapid inhibition, not only of the pathogenic and spoiling species, but also of the flavouring ones. On the other hand, sausages with longer maturation and ripening conducted at a lower temperature are characterized by low acidity and higher numbers of CNCPC resulting in a more aromatic product [12]. CNCPC participate in desirable reactions, such as lipolysis and proteolysis, which influence the aroma, besides being makers of the production and stabilization of colour, reducing nitrates in nitrite [13,14,15].

LAB, therefore, play a primary role in fermented sausages production because they synthesize lactic acid, which causes the lowering of the pH value and the consequent inhibition of pathogenic bacteria and spoilage microorganisms [9,15,16]. Moreover, the acidity accelerates the reduction of the nitrite, and then the process of developing and fixing colour. Finally, LAB release substances, which affect the flavour profile, such as acetic acid, ethanol, acetoin, butanediol, and diacetyl, or having antimicrobial effect, such as bacteriocins [17,18,19].

Over the last 20 years, LAB ecology has been studied by several authors, using both traditional and molecular microbiological techniques. Among them, polymerase chain reaction (PCR) and Denaturing Gradient Gel Electrophoresis (PCR-DGGE) have been extensively used both as a culture-dependent and a culture-independent method [5,20,21,22,23,24]. High-throughput sequencing (HTS) techniques represent a relatively new approach in the way microbiologists address ecology and diversity in different environments, and consequently also in foods. As explained by Franciosa et al. [25], in HTS, mixed nucleic acid molecules from a complex ecosystem can be sequenced, and therefore can lead to a detailed profile of the microbial populations (identified as operational taxonomic units, OTU) present. In the mid-2000s, HTS technologies became ubiquitous in microbial ecology studies, but limitations remain: a) DNA is a chemically stable molecule, which can be found for a long time after the death of a cell; b) quantification of the genera and/or species is only relatively possible. For these reasons this approach cannot be generally applied, but a reasoned choice of method is needed, in particular when ecological studies are made during a relatively short time, and also important is the detection and identification of species whose counts are <10^3^ cfu/g.

The previous studies have identified species belonging to *Lactobacillus* genus as the main ones involved in natural sausage fermentation. The species mainly spotted in this kind of products are *Lactobacillus sakei, Lb. curvatus* and *Lb. plantarum* [26].

Species and strain dynamics and counts in fermented meat products, as demonstrated by many authors, vary according to function of process conditions, particularly NaCl concentration and temperature, which have a direct impact on strain selection, pH, and water activity (A_w_) [27,28,29,30,31]. Other authors illustrated the roles of plant, meat, and ingredients in the selection of a specific microbiota [32,33,34,35].

The role of pork breed and breeding system in influencing LAB ecology during fermented sausages’ ripening has been not yet explored. This is a gap that deserves to be investigated, because they could have direct and undirect effects on the microbial ecology of meat products. In fact, several studies showed the relevant presence of species belonging to the genus *Lactobacillus* in both gut and reproductive tract microbiota of pigs [36,37,38] and demonstrated that breed affects colonic microbiota and immune status since postnatal period (14–49 days) [36,39]. A possible link between the gut microbiota and feed efficiency in pigs was demonstrated by Tan et al. [38], who discovered that, in Landrace pigs, the high-feed conversion rate pigs had a greater abundance of *Lactobacillus* and *Streptococcus* than the low-feed conversion rate pigs, having an evident repercussion on pork meat quality [40]. In parallel, it is now well known that the diet also has a very important influence on the modulation of the intestinal flora, both for humans and for animals, including pigs [41,42]; and the breeding system plays a fundamental role on the type of feeding provided [43]. In the case of the outdoor breeding system, compared to the indoor breeding system, there is a greater variability of nutrients supplied as well as a greater movement of the animals with possible repercussions both in the composition of the intestinal microbiota and on the skin of the animal itself.

Moreover, the microbiological quality of the meat is strictly dependent on the environmental conditions. The meat of healthy and non-fatigued animals is usually sterile, only the lymphatic ganglia and some organs such as liver and spleen can contain microorganisms. However, microorganisms, especially pathogens and/or opportunists, can invade the muscles when the animal is still alive, before slaughter. The causes for this are various and concern, in particular, weakening or stress before slaughter or primary contamination, which can escape the ante-mortem visit: undiagnosed bacteremia and septicemia can be a vehicle for microorganisms deep in the tissues, and psychological stresses can promote the migration of the same from the intestine. The secondary contaminations during all processing phases, remain those to which the cause of the main contamination is due. In particular, during slaughtering, microorganisms present on/in the animal are transferred to the meat, defining its own microbiota [44,45,46].

Thus, the core of our study was to investigate LAB species ecology and dynamics under different experimental conditions. In particular, fermented sausages produced in both artisanal and industrial plants with meat from pigs belonging to three different breeds, and breeding in either indoor or outdoor systems, were investigated.

## 2. Materials and Methods

### 2.1. Fermented Sausages and Sampling Procedures

The experimental design was described in Iacumin et al. [7]. In particular, all pigs (male) were raised under the same conditions on a farm in Friuli Venezia Giulia Region. Animals were at the same age and kept on the same diet and breeding conditions, specifically outdoor or indoor. All the trials were conducted at the same time. Pigs were slaughtered in the same abattoir, on the same day. The carcasses were divided into half-carcasses and evenly distributed between the two processing plants, so that each one had half of each animal available. Two local meat factories in northeaster Italy were selected for the study: plant D (industrial) and plant T (artisanal). The annual sausage production in the industrial plant was approximately 450–500 tons per year. In contrast, the annual sausage production in the artisanal plant was approximately 35–45 tons per year. Starter culture (*Pediococcus pentosaceus*) was used only in the industrial plant. Traditional techniques were used for the production of fermented sausages. The final product was ready for sale after 45–50 (plant D) or 90 (plant T) days. Common ingredients for sausage production included 60 kg pork meat, 40 kg lard, 2.5 kg sodium chloride, 70 g black pepper, and 200 ppm nitrite and nitrate. Sugar was not added for fermentation in plant T, whereas in plant D, 1.5 kg sugar was added. The fermentation mixtures were stuffed into natural casings, and the resulting fresh sausages were 25 cm long and 5 cm in diameter. All of the sausages made in plant D were identical in shape and mass (1 kg each), whereas the sausages made in plant T were not identical in shape, and their masses ranged from 0.8 kg to more than 1 kg each. Ripening parameters were also different in the two plants. The first stage of ripening consisted of 2 days of drying with relative humidity (RH) of 85% at 18–20 °C (plant T) or 22 °C (plant D). The temperature was then decreased to 14 °C (plant T) or 12 °C (plant D) at a rate of 2 °C per day with RH between 60% and 90%. Further ripening was then performed for the rest of the period at those temperatures (14 °C plant T, 12 °C plant D) in storerooms with 65%–85% RH. Plant D had a more effective system to control the environmental temperature and RH than plant T.

In total, 6 batches of products, in triplicate, were monitored in plant D (using industrial production technology) as follows: (i) Cinta Senese meat, indoor breeding system (D-CS-I); (ii) Cinta Senese meat, outdoor breeding system (D-CS-O); (iii) Goland meat, indoor breeding system (D-G-I); (iv) Goland meat, outdoor breeding system (D-G-O); (v) Mora Romagnola meat, indoor breeding system (D-MR-I); and (vi) Mora Romagnola meat, outdoor breeding system (D-MR-O). In plant T (using artisanal production technology), only 4 batches, in triplicate, were monitored: (i) Cinta Senese meat, indoor breeding system (T-CS-I); (ii) Cinta Senese meat, outdoor breeding system (T-CS-O); (iii) Goland meat, indoor breeding system (T-G-I); and (iv) Goland meat, outdoor breeding system (T-G-O).

Every batch of products from each plant was exposed to microbiological analysis. Samplings were performed at 0, 2, 9, 23, 30, 60, and 90 days in plant T and 0, 9, 30, 60, and 90 days in plant D, and three fermented sausages per batches at each sampling point were used for microbiological analysis. A major number of sampling points was applied in plant T, because the fermentation process was slower than in D and this required an extension of the monitoring, in particular at the beginning of the fermentation process.

### 2.2. Microbial Enumeration and Bulk Cell Collection

Serial dilutions of each sample in 0.25X Ringer’s solution (Oxoid, Milan, Italy) were used to inoculate deMan, Rogosa, Sharpe (MRS agar, Oxoid, Milan, Italy) plates, which are widely used to cultivate LAB. Two series of agar plates were inoculated and incubated at 30 °C for 48 h in Jair with AnaeroGen^TM^ 3.5 L kit (Oxoid, Milan, Italy). Portions (0.1 mL) of appropriate dilutions were spread on plates in triplicate. Colonies were counted, and results were calculated as the means of three determinations. After counting, all plates were used for bulk formation as previously described [23]. In brief, a bulk formation was performed using all plates from the serial dilutions (–2 to the last). For each dilution, all colonies grown on the plates’ surface were suspended in 2 mL of quarter strength Ringer’s solution, harvested with a sterile pipette and frozen at −20 °C. To minimise the effects of different concentrations, all suspensions were standardised at 1 unit of optical density (600 nm). Then, 1 mL of the bulk suspension was used for DNA extraction as described below and subjected to molecular analysis.

### 2.3. DNA Extraction from Bulk Cultures

One milliliter of each bulk suspension was centrifuged at 14,000× g for 10 min at 4 °C to pellet the cells, and the pellet was subjected to DNA extraction according to Andrighetto et al. [26]. Briefly: the pellet was added to 30 μL of Lysozyme (0.1 g/mL Lysozyme, 25% Sucrose, Sigma-Aldrich, Milan, Italy) and incubated 30 min at 30 °C for lysis in a Thermomixer (Thermomixer Confort, Eppendorf, Eppendorf AG, Hamburg, Germany).

### 2.4. PCR

Primers P1V1 and P2V1 [24] spanning the V3 region of the 16S rDNA were used in this study. A GC-clamp (5′-CGC CCG CCG CGC CCC GCG CCC GTC CCG CCG CCC CCG CCC G-3) was attached to the 5′ end of primer P1V1 to obtain amplicons to be subjected to DGGE analysis. Amplifications were carried out in a final volume of 25 μL, containing 1 μL (100 ng total) template DNA, 10 mM Tris HCl (pH 8.3), 50 mM KCl, 1.5 mM MgCl_2_, 0.2 mM deoxynucleoside triphosphates (dNTPs), 1.25 U *Taq* polymerase (Invitrogen, Milan, Italy), and 0.2 μM each primer, using the thermal cycler DNA Engine DYAD^TM^ SYSTEM. The amplification cycle included an initial denaturation step at 95 °C for 5 min, followed by 35 series composed by denaturation, performed at 95 °C for 1 min, annealing at 45 °C for 1 min, and extension performed at 72 °C for 1 min. Finally, an extension cycle, performed at 72 °C for 7 min, was added. The PCR products were resolved by agarose (Sigma-Aldrich, Milan, Italy) (2% *w*/*v*) gel electrophoresis at 100 V for 2 h. In each electrophoresis gel, 3 μL of PCR Marker (100 bp) were loaded in the first and in the last well. After running, amplicons were visualized under UV light using the Syngene G: Box Chemi-XX9 (Syngene, Cambridge, United Kindom) and digitally captured by using the software GeneSys version 1.5.7.0 (Syngene, Cambridge, United Kindom).

### 2.5. DGGE Analysis

The DCode universal mutation detection system (Bio-Rad Laboratories S.r.l., Milan, Italy) was used for DGGE analysis. For PCR products obtained with the primers P1V1-GC and P2V1, electrophoresis was performed in a 0.8-mm-thick polyacrylamide gel (8% (wt/vol) acrylamide-bisacrylamide (37.5:1)), with a denaturing gradient from 30% to 50% (100% corresponded to 7 M urea and 40% (wt/vol) formamide) increasing in the direction of the electrophoretic run. Gels were subjected to a constant voltage of 130 V for 3 h and 30 min at 60 °C. After the electrophoresis, gels were stained for 30 min in 1.25X Tris-acetate-EDTA containing 1X SYBR Green (final concentration; Molecular Probes, Milan, Italy). Pictures of the gels were visualized under UV light using the Syngene G: Box Chemi-XX9 (Syngene, Cambridge, United Kindom) and digitally captured by using the software GeneSys version 1.5.7.0 (Syngene, Cambridge, United Kindom).

A reference pattern was established consisting of amplicons from 4 different bacterial species: *Lactobacillus brevis* (DSMZ 20054), *Lb. casei* (DSMZ 20011), *Lb. curvatus* (DSMZ 6179) and *Lb. sakei* (DSMZ 6333). By including this standard reference pattern three times on each DGGE gel, resulting DGGE fingerprint band profiles from the different sausages were digitally normalised using Gel Compare 4.1 software Version 4.1 (Applied Maths, Kortrijk, Belgium). Additionally, this reference pattern was used to obtain a preliminary identification of the species. When possible, almost three bands migrating the same position in every single gel were excised and subjected to sequencing and sequence analysis to confirm the preliminary identification and to identify amplicons, which did not correspond to those of the reference strains used. DGGE analyses were performed at least twice.

### 2.6. Sequence Analysis of DGGE Bands

Blocks of polyacrylamide gels containing selected DGGE bands were excised with gel cutting tips. Blocks were then transferred to 100 μL sterile water, and the DNA in the bands was left to diffuse overnight at 4 °C. Two microliters of the eluted DNA were used for re-amplification, and PCR products generated with a GC-clamped primer were verified by DGGE. Only products migrating as a single band and in the same position of the control were amplified, as described above, with the primer without the GC clamp. Products were then cloned into the pGEM-T Easy vector (Promega, Milan, Italy) following the manufacturer’s instructions. Clones were checked as described above (co-migration with control), and the inserts in appropriate clones were sequenced at a commercial facility (Eurofins, Edersberg, Germany). Sequence comparisons were performed in GenBank using the Blast program version 2.2.18 (https://blast.ncbi.nlm.nih.gov/Blast.cgi) [47].

### 2.7. Statistical Analysis

The statistical tests were performed using R software, vers. 3.4.0 (R core team, 2017, Vienna, Austria). Normality of data distribution and homoscedasticity were tested using Shapiro–Wilk and Levene test, respectively. Data were assessed within the plant. The LAB count in the plant D was analysed with a two-way ANOVA model, with experimental group (batches) and ripening time as factors. Also, the interaction experimental group × ripening time was considered. For multiple comparisons, the P-values were adjusted using the Holm method, and, when appropriate, the White-corrected P-values [48] were considered. The same model, but with the Scheirer–Ray–Hare test for non-parametric data [49] was used for LAB count in the plant T. Multiple comparisons were performed using Mann–Whitney U test, and the P-values were adjusted using the Holm method.

As a result of the “bulks”, the molecular analysis of different fingerprints was performed. Analysis of the patterns and their corresponding dilutions give information about the dominant species occurring and also allows to ascertain the concentration of every single species found in the DGGE profile of the original sample. However, these concentration values are not properly quantitative, they are ordinal value and in statistical analysis they have to be treated as qualitative parameters. For this reason, and to avoid a subjective conversion of the data (from bands in numeric values), a direct cluster analysis from the obtained fingerprints was chosen [7].

The different profiles of each dilution from the same sample were previously joined to get a total sample profile. The profiles of the different times are not treated in a bulk for each sample, but one by one. Pictures of the gels were analysed by using the pattern analysis software package Gel Compare Version 4.1 (Applied Maths, Kortrijk, Belgium). Calculations of similarities in band profiles were based on Pearson (correlation coefficient at 53%) and Dice (correlation coefficient 60%) product-moment correlation coefficients. Dendrograms were obtained via the unweighted pair group method using an arithmetic average (UPGMA) clustering algorithm [50]. Two different analyses, using different correlation coefficients (Pearson and Dice), were applied to verify the strength of the results.

## 3. Results and Discussion

### 3.1. LAB Counts

LAB counts in the different trials and at the different sampling points corresponding to the different fermented sausages production steps were shown in Table 1. Fermentations in plant T were characterised by lower initial LAB counts compared to that in plant D, where *P. pentosaceus* was added as starter culture (*P* < 0.01).

In plant T, the statistical analyses highlighted a significant effect of ripening time (*P* < 0.01), but not of experimental group (*P* > 0.05) on LAB count. Also, the interaction *ripening time × experimental group* did not reach a level of significance (*P* > 0.05). LAB count increased from t0 to t23 (*P* < 0.05), after which counts remained stable till T30 (*P* > 0.05) and then decreased at t90 (*P* < 0.05), where LAB count was similar to that observed at t9 (*P* > 0.05). LAB counts slightly decresed at t90 (*P* < 0.05), where LAB count was similar to that observed at t9 (*P* > 0.05). At the same time, this difference was not significant compared to LAB count at t60. From a microbiological point of view, this can be explained by the fact that at t90 the physic-chemical characteristics of the products, such as the combined effect of pH (5.32 ± 0.13, data not shown), activity water (Aw, 0.925 ± 0.024, data not shown) and the absence of sugars led to a progressive slow inactivation of LAB.

In plant D, LAB count was affected both by experimental group (*P* < 0.01) and by ripening time (*P* < 0.01). Moreover, also the interaction *experimental group × ripening time* was significant (*P* < 0.01). It means that the main effect of a factor can be interpreted only at each level of the other factor. Therefore, in Table 1, the interaction from the perspective of the experimental group is represented in a row, and the interaction from the perspective of ripening time is represented in a column. The first of these shows that LAB count increased from t9 to t30 (*P* < 0.05) and, then, remained stable from t30 to t90 (*P* > 0.05) for the sausages produced with Goland breed, irrespectively of the husbandry system adopted. Furthermore, in the D-MR-I group, LAB count remained stable from t30 to t90 (*P* > 0.05), but, in this group, LAB count started to increase already from t0 to t9 (*P* < 0.05). The D-CS-I and D-MR-O groups showed LAB count that increased from t0 to t9 (*P* < 0.05), but decreased from t60 to t90 (*P* < 0.05). Conversely, LAB count in D-CS-O decreased from t0 to t9 (*P* < 0.05) and the value recorded at t90 was similar to that shown at t9 (*P* > 0.05). Considering the results about the interaction *experimental group × ripening time* from the perspective of the ripening time, at t0, the lowest LAB count values were observed in sausages produced with the Goland and Cinta Senese breed indoor-housed (*P* < 0.05), conversely, the higher values were recorded in the sausages produced with the same breeds, but with outdoor-housed animals (*P* < 0.05). Lower differences between experimental groups were found in the other ripening times with LAB count that was lower in D-G-I than D-MR-O (*P* < 0.05) at t30, and in D-CS-O than in D-CS-I (*P* < 0.05) and D-MR-O (*P* < 0.05) at t60. Moreover, sausages produced with Cinta Senese breed had lower LAB count that those observed in D-G-O group at t90 (*P* < 0.05).

**Figure 1:**
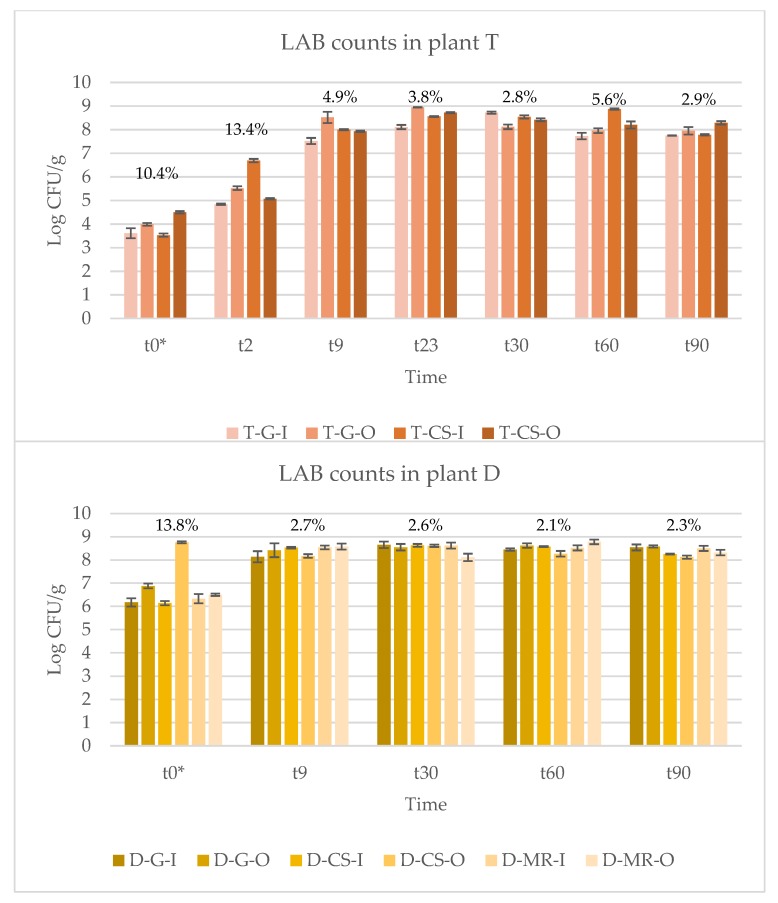
LAB development during ripening in the different trials. Industrial plant (D) and traditional plant (T) whose meats derived from pigs of three breeds, Goland (G), Cinta Senese (CS), and Mora Romagnola (MR) that were indoor (I) or outdoor (O) housed. The coefficient of variation among trials per time is shown above histograms.

The comparison along the time is not the effect under study, but it allowed to verify if there were deviations in the correct development of fermentation. If this had happened, this could have been attributed to the different composition (e.g., intramuscular fat, type of fat.) of the meat due to the breed. In Figure 1 it can be observed that, apart from t0, the coefficients of variation among the trials per each time is higher in plant T than in plant D. This can be attributed to the different procedures and technologies applied in the two plants. The industrial plant (D), using exactly the same procedures at each production phase, as well as an optimized protocol of drying and ripening in chambers with a more accurate system to control humidity and temperature, resulted in a major standardization of the microbial fermentation and, consequently, of the final product. On the other hand, although in the artisanal plant (T) there is a higher coefficient of variability, it is possible to note that fermentation has progressed flawlessly. In this case, no microbial starters have been added, but nevertheless the production technology has allowed the correct development of the indigenous LAB, which in nine days have reached the same values as those inoculated in plant D. Finally, looking at day 0, it seems that the counts of the trials performed using pork meat from outdoor breeding system were significantly higher than those of the indoor breeding system. Probably the outdoor breeding system has brought the pigs to smear/soil more than the indoor breeding system, and therefore to increase the microbial load of the skin, which during the slaughter has favoured a greater contamination of the carcass.

### 3.2. Dynamics of the Detected Species

**Figure 2:**
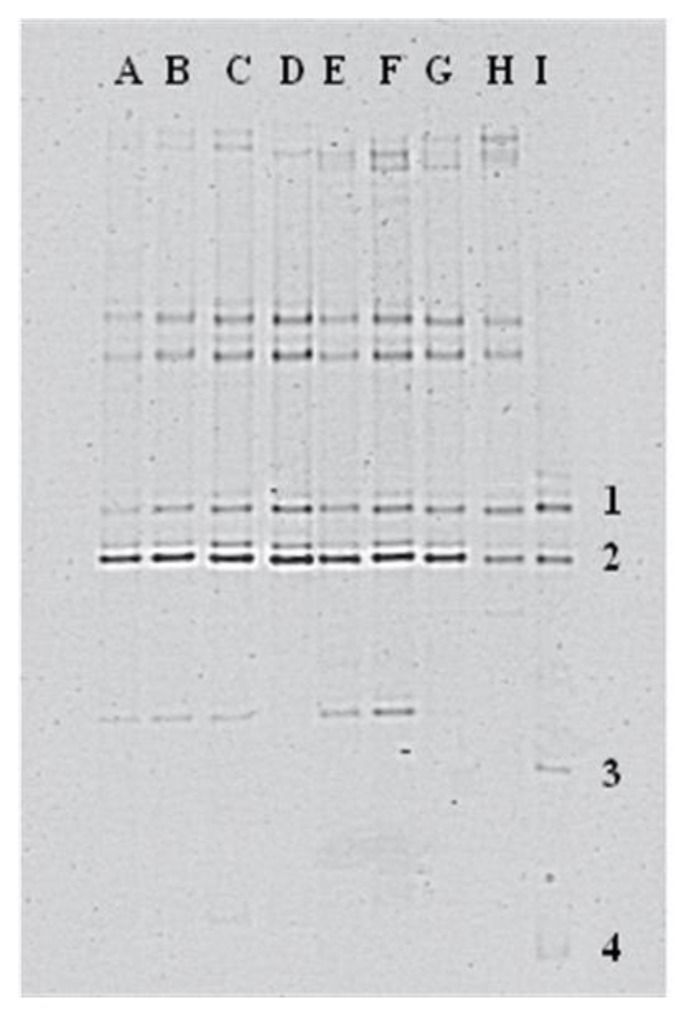
Examples of PCR-DGGE fingerprints obtained from the bulk suspensions. Lines A, B, C, D, E, F, G correspond to the samples coming from samples T-CS-I. Line I correspond to the standard used as a control: 1—*Lb. curvatus*, DSM 20019; 2—*Lb. sakei*, DSM 6333; 3—*Lb. brevis*, DSM 20054; 4—*Lb. casei*, DSM 20011.

In order to obtain information regarding the species involved in the fermentation and their relative concentration, DGGE fingerprints were obtained from cells collected in bulk from the count plates for each dilution, after colony enumeration. From the DGGE fingerprints (Figure 2), the following species, as responsible for the fermentation of sausages, were detected: *Lb. sakei*, *Lb. casei*, *Lb. cuvatus*, *Lb. graminis*, *Lactococcus garviae*, *Leuconostoc mesenteoides*, and *Pediococcus pentosaceus*.

In particular, *Lb. sakei* constituted the predominant specie over the monitored period. In this study, it has been therefore confirmed that *Lb. sakei* remains the overriding species in fermented meat products, as demonstrated by several authors, who reported percentages of isolation starting from 42% in Italian-Greek suasages, increasing to a percentage of 76%–89% in Spanish sausages and reaching the 100% in French sausages [21,27,51,52,53,54].

In samples from the industrial plant (plant D, Table 2) a limited number of species, ranging from two to four, were detected in the different trials. *Lb. sakei* and *P. pentosaceus* were present in all monitored fermentations and showed the highest counts during the entire fermentation process. Considering that in these trials *P. pentosaceus* was inoculated as starter culture at a level of about 10^6^ CFU/g, the results highlighted the capability of *Lb. sakei* to dominate in this peculiar ecological niche being the more adaptable species at the specific environmental conditions of fermented meats. In fact, although from the beginning of fermentation high counts of *P. pentosaceus* were present, *Lb. sakei* was able to compete and achieve comparable concentrations, remaining almost stable throughout the fermentation process. The development of *Lb. sakei* was favoured by the temperature of the first stage of ripening (22 °C), which is more adapted to *Lb. sakei* than *P. pentosaceus*. Indeed, the choice to use a first ripening temperature lower than the optimal temperature for *P. pentosaceous* growth was determined by the aim of reducing the rate of acidification by this species, in order to obtain a more flavourful product.

Other than these two species, also *Lb. curvatus* was found, confirming to be one of the most recurrently species, suggesting that, in association with *Lb. sakei*, it predominantly leads meat fermentations [33,55,56,57,58]. In fact, *Lb. curvatus* was present during the ripening of industrial products, except for those obtained from Cinta Senese and Mora Romagnola indoor breeding system productions (D-CS-I, D-MR-I).

In the artisanal products (plant T, Table 3), this species had a more constant trade, from the stuffing to the end of ripening, where it amounted to the highest dilutions (10^−6^–10^−7^). Instead, in the industrial productions (D), it was constantly founded in Goland outdoor, Cinta Senese indoor and Mora Romagnola outdoor breeding system products (D-G-O, D-CS-I, D-MR-O), whereas in the other cases *Lb. sakei* was only detected at the end of the ripening.

Considering the artisanal plant, *Lb. sakei* and *Lb. curvatus* were detected in all the monitored products, especially after the ninth day of ripening, always at the higher dilutions (10^−5^–10^−7^), once more confirming their dominant role also in spontaneous fermentations. *Lb. casei* was the least common species identified in the sausages from both the plants, but it appeared at high concentration at 90 days for the industrial D-G-I trial, and only from zero to nine days in the artisanal spontaneous fermentation T-G-I. Considering the relevant aspects related to the probiotic characteristics of this specie, their presence as spontaneous microflora in this products supports gives clear evidence that this species could be used as starter culture to provide the market with a new nutritional alternative that increases the variety of existing probiotic products, as well as increasing the added value of sausages [59,60].

Other species were detected during the process, but they were involved only in some of the considered productions. The equilibrium among the different species as well as the presence or absence of some of them are responsible for the production of different aroma characteristics in the final product; characteristics that are even more evident in the comparison of artisanal rather than industrial products.

*Leuconostoc mesenteroides* was identified in each of the artisanal productions, till the 60^th^ day of ripening. *Lactococcus garviae* was detected in each monitored sausage, except for the Goland outdoor breeding system products (T-G-O). *Lb. casei, Lb. graminis*, and *Pediococcus pentosaceus* were only found in the Goland indoor breeding system products (T-G-I).

*Lb. curvatus* was constantly participating in artisanal productions (T), especially in sausages obtained from Cinta Senese breed, reaching the highest dilutions (10^−7^) at the end of ripening. Products from Goland breed showed some differences depending on the breeding system: in T-G-I, *Lb. curvatus* was always detected during ripening, whereas in T-G-O, this species was founded only in the first step of the process. Fermented sausages from the industrial plan (D) showed an irregular presence of *Lb. curvatus* during ripenings. In some cases, it was absent, in other ones, it was detected only at the last steps of ripening, and in other trials, this species was found all long the production process. *Pediococcus pentosaceus* had a specific role in industrial sausage production (D) due to its use as a microbial starter; indeed, in this case, this species was detected, from the stuffing to the end of the ripening, at the greater dilutions. In the artisanal productions (T), *P. pentosaceus* was founded only in sausages obtained by Goland pork breed at the sampling point named t30. *Leuconostoc mesenteroides* was observed only in the artisanal products (T), with an irregular presence. In samples from the outdoor breeding system, this species was detected at the moment of stuffing, in low dilutions (10^−2^–10^−3^); in samples from Cinta senese indoor breeding system (T-CS-I), *L. mesenteroides* was present till t30 sampling time, in increasing dilutions (from 10^−2^ to 10^−7^); in sausages from Goland indoor breeding system (T-G-I), this species appeared from t23 to t60 sampling points, in high dilutions (10^−6^–10^−7^). *Lb. casei* was detected in both plants (T, D), but only in products obtained from Goland indoor breeding system, during the first step of ripening. *Lactococcus garviae* was detected in artisanal products (T) deriving from indoor breeding, at the first sampling times and in the highest dilutions (10^−7^). *Lactococcus garvieae* was rarely associated to fermented sausages [15,51] and it has been classified as an emerging pathogen found in animals (cows, buffalos, farmed fish), as well as in human clinical isolates. Since then, many papers stated that this bacterium is a human gastrointestinal commensal or transient bacterium, which can cause a variety of infections [61]. *Lb. graminis* was present only in artisanal fermented sausages (T), from Goland indoor breeding system, during the middle period of ripening (t2–t23), in high dilutions (10^−6^–10^−7^). This species was at first isolated at the end fermentation of grass silage. Noteworthy that, this heterofermentative species, which is not frequently isolated from meat products, has a low DNA-DNA homology value with the type strains of *L. sakei* and *L. curvatus*, but they were similar with respect to D(-)- and L(+)- lactate formation, Rf-values of the D- and L-LDH, G+C content of the DNA and the L-Lys-D-Asp murein type [62].

### 3.3. Fingerprint Analyses

**Figure 3:**
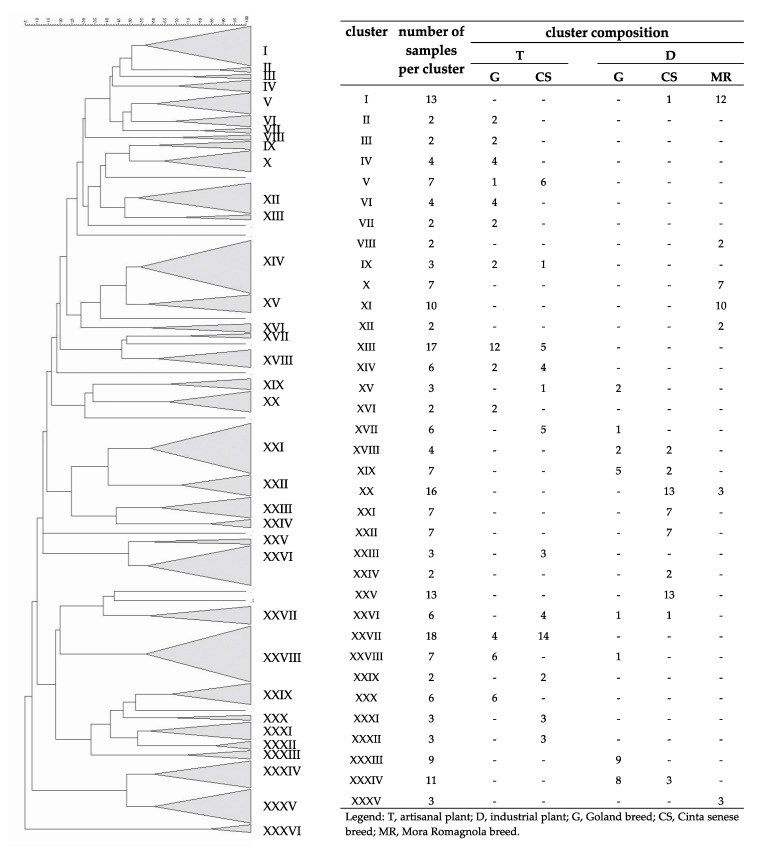
Cluster analysis using Pearson product–moment correlation coefficients and unweighted pair group method using an arithmetic average (UPGMA) of the profiles obtained from the fingerprinting analysis of the different fermentations. A similarity coefficient of 53% was arbitrarily chosen. Identified clusters are indicated with roman numerals. Cluster composition is defined in the table within the figure.

**Figure 4:**
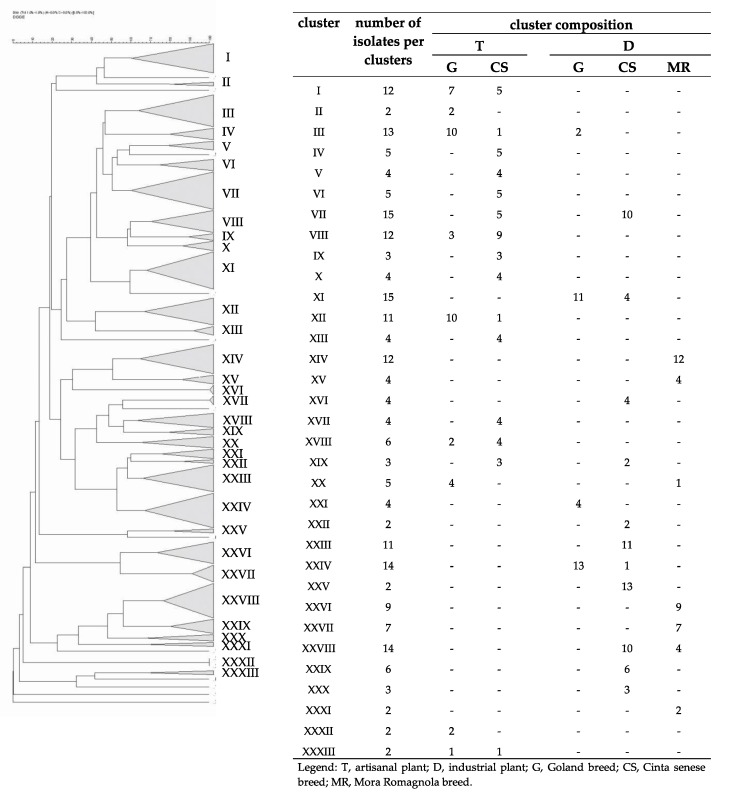
Cluster analysis using Dice product–moment correlation coefficients and unweighted pair group method using an arithmetic average (UPGMA) of the profiles obtained from the fingerprinting analysis of the different fermentations. A similarity coefficient of 60% was arbitrarily chosen. Identified clusters are indicated with roman numerals. Cluster composition is defined in the table within the figure.

Statistical analysis was also applied to the DNA fingerprints obtained by PCR-DGGE. The results of the cluster analysis using the Pearson product-moment correlation coefficient were shown in Figure 3. A similarity coefficient of 53% was arbitrarily selected. As shown (Figure 3), a total of 35 clusters were discerned. Except for clusters XV, XVII, XXVII and XXIX, all the others grouped samples produced in the same plant, clearly suggesting that they were plant-specific clusters. Half of the groups included only two, three, or four samples, the others are composed of six or more samples. The most relevant clusters were the number XIII (17 fingerprints, plant T), XX (16 fingerprints, plant D), XXV (13 fingerprints, plant D) and XXVII (18 fingerprints, plant T). Furthermore, 22 clusters were found to be also breed-specific. Only nine samples did not belong to any cluster. Subsequently, a different cluster analysis based on the Dice correlation coefficient (60% coefficient of similarity) was performed, and the results are presented in Figure 4. The second analysis confirmed the previous results: 20 clusters of 33 resulted to be plant-specific, 15 of them included two, three, or four samples and the others had five or more samples. The most relevant clusters are the number XI (15 fingerprints, plant D), XXIV (14 fingerprints, plant D) and XXVIII (14 fingerprints, plant D). This analysis confirmed also the presence of breed-specific clusters (22 of 33) and 12 samples did not belong to any cluster.

The congruence of the data obtained with the use of both Pearson and Dice product-moment-correlation coefficients proves the significance of the result, demonstrating the existence of plant- and breed-specificity among the monitored fermentations. If the plant effect could be simply explained as an environmental selection during the time of the microflora, which forms a biofilm on structures and equipment, the breed effect could result from the different chemical-physical characteristics of the raw meats, more precisely the type and quantity of intramuscular fat, which are strictly correlated to pork breed. Actually, during grinding and/or mixing, the rise in temperature due to friction can cause the melting of low melting point fats, with consequent difficulties in the dehydration process during drying and ripening, leading to irregular changes in water activity (Aw) that influence the microbial species equilibrium. Finally, most of the breed-specific clusters, obtained by both the correlation coefficients, showed homogeneous samples also concerning the different breeding systems. Therefore, it’s possible to say that the microbial ecology is influenced also by the breeding system, as speculated in the introduction. However, it is possible that other factors may intervene and change the microflora characterizing the animal and, consequently, the meat. In our experiment, for example, seasonality was not taken into account, to avoid adding too many variables. It should be considered, however, that, among others, humidity and environmental temperature can influence both the environmental microflora and the state of health of the animal, with consequent modification of the intestinal microflora, leading to variations of the isolated species. Furthermore, it is well known that small variations can always be present, considering the differences between the autochthonous and allochthonous microflora, but the former will always have a character of stability, due to the fact that being more adapted to that particular environment, it will also be more incisive than the allochthonous ones. These results confirm the data from previous studies, which stated that the production technologies applied in a specific production plant and the pork breed select a characteristic and specific microbial ecology [6,7,34,63].

## Figures and Tables

**Table 1:** Mean ± standard deviation of Lactic acid bacteria (LAB) colony counts, expressed as Log CFU/g, of sausages at different ripening days (t) and produced in two plants, industrial (D) and traditional (T) whose meats derived from pigs of three breeds, Goland (G), Cinta Senese (CS), and Mora Romagnola (MR) that were indoor (I) or outdoor (O) housed.

Processing Time							
	t0 *	t2	t9	t23	t30	t60	t90
**T-G-I**	3.61 ± 0.21	4.84 ± 0.03	7.52 ± 0.13	8.11 ± 0.09	8.72 ± 0.05	7.73 ± 0.14	7.75 ± 0.01
**T-G-O**	3.99 ± 0.06	5.52 ± 0.08	8.52 ± 0.24	8.95 ± 0.01	8.12 ± 0.10	7.96 ± 0.10	7.95 ± 0.16
**T-CS-I**	3.53 ± 0.07	6.69 ± 0.07	8.00 ± 0.02	8.56 ± 0.02	8.54 ± 0.07	8.87 ± 0.03	7.78 ± 0.03
**T-CS-O**	4.50 ± 0.05	5.07 ± 0.03	7.93 ± 0.03	8.72 ± 0.03	8.42 ± 0.06	8.20 ± 0.15	8.29 ± 0.07
**Mean**	3.91 ± 0.41 ^α^	5.53 ± 0.74 ^β^	7.99 ± 0.39 ^γ^	8.62 ± 0.33 ^δ^	8.45 ± 0.24 ^δ^	8.19 ± 0.46 ^γδ^	7.94 ± 0.23 ^γ^
**D-G-I**	6.17 ± 0.18 ^Aα^	n.d.	8.14 ± 0.24 ^α^	8.47 ± 0.12 ^β^	8.65 ± 0.14 ^Aβ^	8.45 ± 0.05 ^ABβ^	8.54 ± 0.13 ^ABβ^
**D-G-O**	6.88 ± 0.10 ^Cα^	n.d.	8.42 ± 0.30 ^α^	n.d.	8.55 ± 0.14 ^ABβ^	8.62 ± 0.10 ^ABβ^	8.58 ± 0.05 ^Bβ^
**D-CS-I**	6.14 ± 0.09 ^Aα^	n.d.	8.53 ± 0.04 ^γ^	n.d.	8.63 ± 0.06 ^ABγ^	8.58 ± 0.02 ^Bγ^	8.25 ± 0.02 ^Bβ^
**D-CS-O**	8.76 ± 0.04 ^Dα^	n.d.	8.17 ± 0.08 ^β^	n.d.	8.61 ± 0.05 ^ABγ^	8.27 ± 0.12 ^Aβ^	8.12 ± 0.07 ^Aβ^
**D-MR-I**	6.33 ± 0.20 ^ABα^	n.d.	8.54 ± 0.08 ^β^	n.d.	8.62 ± 0.13 ^ABβ^	8.52 ± 0.11 ^ABβ^	8.50 ± 0.11 ^ABβ^
**D-MR-O**	6.50 ± 0.05 ^Bα^	n.d.	8.58 ± 0.13 ^βγ^	n.d.	8.11 ± 0.16 ^Bβ^	8.78 ± 0.10 ^Bγ^	8.32 ± 0.12 ^ABβ^
**Mean**	6.80 ± 0.94 ^α^		8.39 ± 0.23 ^β^	8.47 ± 0.12 ^β^	8.53 ± 0.22 ^β^	8.53 ± 0.18 ^β^	8.38 ± 0.19 ^β^

Legend: ^A, B, C, D^: *P* < 0.05 within plant and column; ^α, β, γ, δ^: *P* < 0.05 within plant and row; n.d.: not done; *t: days.

**Table 2:** Plate dilutions at which each identified species were detected per sampling point from the industrial plant (D).

Identified Species (NCBI Accession Number)	t0	t9	t30	t60	t90
	Plate Dilution at Which the Identified Species were Detected				
**D-G-I**					
*Lactobacillus sakei (NR_113821.1)*	10^−2^	n.d.	n.d.	n.d.	10^−6^
*Lactobacillus curvatus (NR_113334.1)*	n.d.	n.d.	n.d.	n.d.	10^−7^
*Lactobacillus casei (NR_041893.1)*	n.d.	n.d.	10^7^	n.d.	10^−7^
*Pediocossus pentosaceus (NR_042058.1)*	10^−7^	10^−7^	10^−7^	10^−7^	10^−7^
**D-G-O**					
*Lactobacillus sakei (NR_113821.1)*	10^−3^	10^−6^	10^−7^	10^−7^	10^−7^
*Lactobacillus curvatus (NR_113334.1)*	n.d.	n.d.	10^−6^	10^−6^	10^−6^
*Pediocossus pentosaceus (NR_042058.1)*	10^−6^	10^−6^	10^−7^	10^−7^	10^−7^
**D-CS-I**					
*Lactobacillus sakei (NR_113821.1)*	10^−2^	10^−6^	10^−7^	10^−7^	10^−6^
*Pediocossus pentosaceus (NR_042058.1)*	10^−5^	10^−7^	10^−7^	10^−7^	10^−7^
**D-CS-O**					
*Lactobacillus sakei (NR_113821.1)*	n.d.	n.d.	n.d.	10^−5^	10^−4^
*Lactobacillus curvatus (NR_113334.1)*	n.d.	n.d.	n.d.	10^−5^	10^−5^
*Pediocossus pentosaceus (NR_042058.1)*	10^−6^	10^−7^	10^−7^	10^−6^	10^−7^
**D-MR-I**					
*Lactobacillus sakei (NR_113821.1)*	n.d.	n.d.	n.d.	10^−6^	10^−4^
*Pediocossus pentosaceus (NR_042058.1)*	10^−5^	10^−7^	10^−7^	10^−7^	10^−6^
**D-MR-O**					
*Lactobacillus sakei (NR_113821.1)*	n.d.	10^−7^	10^−7^	10^−7^	10^−7^
*Lactobacillus curvatus (NR_113334.1)*	n.d.	10^−7^	10^−7^	10^−7^	10^−7^
*Pediocossus pentosaceus (NR_042058.1)*	10^−6^	10^−7^	10^−7^	10^−7^	10^−7^

n.d.: not detected; D-CS-I: Industrial plant—Cinta Senese meat—indoor breeding system; D-CS-O: Industrial plant—Cinta Senese meat—outdoor breeding system; D-G-I: Industrial plant—Goland meat—indoor breeding system; D-G-O: Industrial plant—Goland meat—outdoor breeding system.

**Table 3:** Plate dilutions at which each identified species were detected per sampling point from the artisanal plant (T).

Identified Species (NCBI Accession Number)	t0	t2	t9	t23	t30	t60	t90
	Plate Dilution at Which the Identified Species were Detected						
**T-G-I**							
*Lactobacillus sakei (NR_113821.1)*	10^−2^	10^−5^	10^−5^	10^−5^	10^−6^	10^−6^	n.d.
*Lactobacillus curvatus (NR_113334.1)*	10^−2^	10^−4^	10^−5^	10^−5^	10^−6^	10^−6^	10^−6^
*Lactobacillus casei (NR_041893.1)*	10^−2^	10^−4^	10^−6^	n.d.	n.d.	n.d.	n.d.
*Lactobacillus graminis (NR_042438.1)*	n.d.	n.d.	n.d.	n.d.	n.d.	n.d.	10^−6^
*Lactococcus garviae (KU898985.1)*	n.d.	10^−3^	10^−6^	10^−7^	n.d.	n.d.	n.d.
*Leuconostoc mesenteroides (DQ297412.1)*	n.d.	n.d.	n.d.	10^−7^	10^−6^	10^−6^	n.d.
*Pediocossus pentosaceus (NR_042058.1)*	n.d.	n.d.	n.d.	10^−5^	10^−5^	n.d.	n.d.
**T-G-O**							
*Lactobacillus sakei (NR_113821.1)*	10^−2^	10^−4^	10^−7^	10^−7^	10^−6^	10^−6^	10^−6^
*Lactobacillus curvatus (NR_113334.1)*	10^−2^	10^−4^	10^−7^	10^−7^	10^−6^	n.d.	n.d.
*Leuconostoc mesenteroides (DQ297412.1)*	10^2^	n.d.	n.d.	n.d.	n.d.	n.d.	n.d.
**T-CS-I**							
*Lactobacillus sakei (NR_113821.1)*	10^−2^	10^−6^	10^−6^	10^−7^	10^−7^	10^−7^	10^−7^
*Lactobacillus curvatus (NR_113334.1)*	n.d.	10^−6^	10^−6^	10^−7^	10^−7^	10^−7^	10^−7^
*Lactococcus garviae (KU898985.1)*	10^−3^	10^−4^	n.d.	n.d.	n.d.	n.d.	n.d.
*Leuconostoc mesenteroides (DQ297412.1)*	10^−2^	10^−6^	n.d.	10^−5^	10^−7^	n.d.	n.d.
**T-CS-O**							
*Lactobacillus sakei (NR_113821.1)*	n.d.	10^−3^	10^−7^	10^−7^	10^−7^	10^−6^	10^−7^
*Lactobacillus curvatus (NR_113334.1)*	n.d.	10^−3^	10^−7^	10^−7^	10^−7^	10^−6^	10^−7^
*Lactococcus garviae (KU898985.1)*	n.d.	10^−3^	n.d.	n.d.	n.d.	n.d.	n.d.
*Leuconostoc mesenteroides (DQ297412.1)*	10^−3^	n.d.	n.d.	n.d.	n.d.	n.d.	n.d.

n.d.: not detected; D-CS-I: Industrial plant—Cinta Senese meat—indoor breeding system; D-CS-O: Industrial plant—Cinta Senese meat—outdoor breeding system; D-G-I: Industrial plant—Goland meat—indoor breeding system; D-G-O: Industrial plant—Goland meat—outdoor breeding system; D-MR-I: Industrial plant—Mora Romagnola meat—indoor breeding system; D-MR-O: Industrial plant—Mora Romagnola meat, outdoor breeding system.
